# Cervical Necrotizing Fasciitis Caused by Dental Extraction

**DOI:** 10.1155/2016/1674153

**Published:** 2016-06-08

**Authors:** José Alcides Arruda, Eugênia Figueiredo, Pâmella Álvares, Luciano Silva, Leorik Silva, Antônio Caubi, Marcia Silveira, Ana Paula Sobral

**Affiliations:** ^1^Faculdade de Odontologia de Pernambuco, Universidade de Pernambuco, Avenida General Newton Cavalcante, 1650 Aldeia dos Camarás, 54753-020 Camaragibe, PE, Brazil; ^2^Hospital da Restauração, Avenida Governador Agamenon Magalhães, S/N, Derby, 52010-040 Recife, PE, Brazil; ^3^Universidade Federal do Rio Grande do Norte, Campus Universitário Lagoa Nova, P.O. Box 1524, 59078-970 Natal, RN, Brazil

## Abstract

Cervical necrotizing fasciitis is an unusual infection characterized by necrosis of the subcutaneous tissue and fascial layers. Risk factors for the development of necrotizing fasciitis include diabetes mellitus, chronic renal disease, peripheral vascular disease, malnutrition, advanced age, obesity, alcohol abuse, intravenous drug use, surgery, and ischemic ulcers. This report presents a case of necrotizing fasciitis in the cervical area caused by dental extraction in a 73-year-old woman. Cervical necrotizing fasciitis in geriatric patient is rare, and even when establishing the diagnosis and having it timely treated, the patient can suffer irreversible damage or even death. Clinical manifestations in the head and neck usually have an acute onset characterized by severe pain, swelling, redness, erythema, presence of necrotic tissue, and in severe cases obstruction of the upper airways. Therefore, the presentation of this clinical case can serve as guidance to dentists as a precaution to maintain an aseptic chain and be aware of the clinical condition of older patients and the systemic conditions that may increase the risk of infections.

## 1. Introduction

Necrotizing fasciitis (NF) is a potentially fatal infection generally characterized by a rapidly progressive process of necrosis of the subcutaneous tissues and muscle fascial layers. The most likely cause of the disease is a vascular obliteration with microthrombosis around the locus of infection, accompanied by acute inflammation of the subcutaneous tissue and swelling of the underlying tissues; with the progression there is no intravascular coagulation in place and the infected tissue becomes necrotic. Moreover, it is believed that the lack of specific antigens of group A streptococcus is a predisposing factor for the development of this disease [1]. NF in the cervical area caused by dental extraction in a geriatric patient is rare and only few cases in the medical literature in English are reported.

The primary sites of these infections in the head and neck are teeth, tonsils, or traumatic wounds [1]. To establish the diagnosis, areas of necrotic tissue surrounding the underlying fabric and the fascial spaces should be clinically observed. Clinical manifestations usually have acute onset and are characterized by intense algic pain, swelling, redness, erythema, the presence of necrotic tissue, presence of palpable crepitus due to subcutaneous gas, grey, foul-smelling “dishwater” exudate, and in severe cases obstruction of the upper airways [2, 3].

This report presents a case of NF in the cervical area caused by dental extraction in a 73-year-old woman.

## 2. Case Presentation

**Figure 1:**
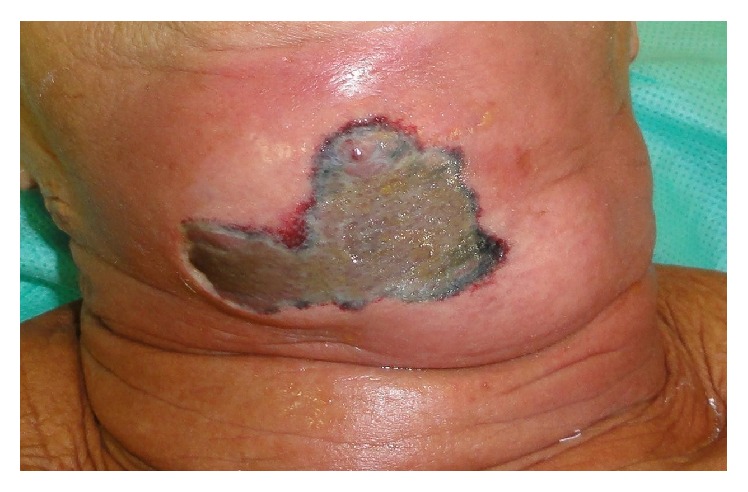
The extraoral physical examination revealed extensive necrotic tissue in the cervical area.

**Figure 2:**
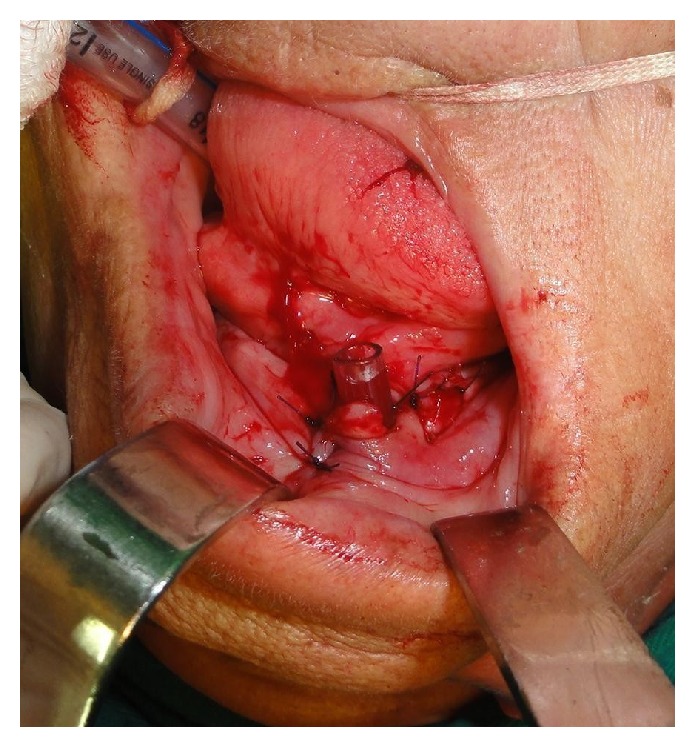
Installation of a Pen Rose irrigation drain.

**Figure 3:**
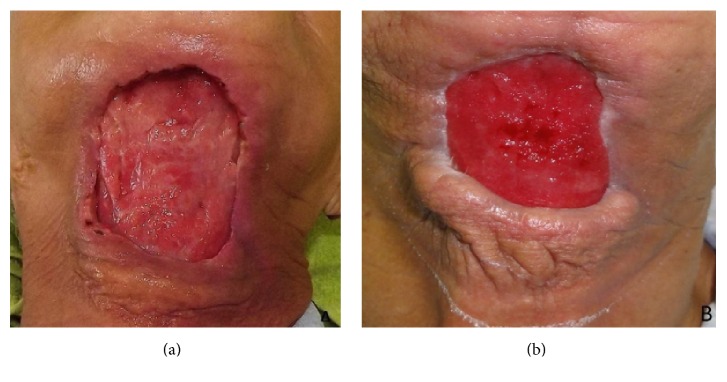
(a) After seven days and (b) after 21 days, dehiscence of the wound edges was observed.

**Figure 4:**
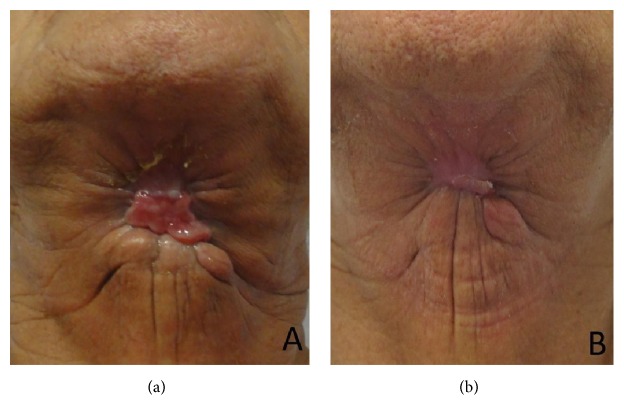
(a) After 30 days and (b) after seven weeks using silver alginate, a favorable healing was observed.

A 73-year-old woman attended at the emergency ward of Universidade de Pernambuco's Hospital da Restauração, Brazil, complaining of severe pain in the cervical area. During the anamnesis the patient reported extraction of the lower right lateral incisor six days priorly. The extraoral physical examination revealed extensive necrotic tissue in the cervical area, fetid odor, hyperemia, and edema (Figure 1). Respiratory rate and blood pressure changes, however, were not observed. Culture was performed with antibiotic susceptibility of the affected region. Biochemical tests revealed a blood count showing leukocytosis (22,000 mm^3^) and a slightly increased glycemic index (144 mg/dL). Clinical diagnosis of NF was established. Preoperative antibiotic therapy was administered with Metronidazole 500 mg and Rocefin 1 g. The patient was submitted to surgical removal of necrotic tissue, debridement of the surrounding tissues, installation of a Pen Rose irrigation drain (physiologic solution 0.9%), and intravenous administration of Meropenem 500 mg for 10 days (Figure 2). After seven days, dehiscence of the wound edges was observed, a new surgical debridement was carried out, and occlusive dressing with silver alginate was applied and exchanged every 48 hours for 21 days (Figures 3(a) and 3(b)). After 30 days, the patient was discharged (Figure 4(a)), though complete repair of the surgical wound was not observed, as that only happened seven weeks after the operation by secondary intention (Figure 4(b)).

## 3. Discussion

The NF predominantly affects the tissues of the abdominal wall, the peritoneum, and extremities, being quite rare (approximately 3-4% of all NF cases) in the head and neck. The severity of this disease depends on the etiology, anatomical site, tissue depth, type of bacteria, and general condition of the patient [4–6]. NF usually occurs secondarily to dental or gingival infections. Whitesides et al. [7] observed that the second and third molars were commonly the cause of this injury. However, any spread of microorganisms that causes some deep infection in the adjacent tissues of the neck can cause NF [8]. Extraction prior to the NF might be raised as a cause of breaking the aseptic chain during this surgical procedure and disrupting the systemic condition of the patient, and the infection in the present case may have begun this way, as the patient reported an extraction of the lower right lateral incisor six days priorly.

The age of the patient may have been triggering, despite the fact that NF can occur in all ages; however, Maria and Rajnikanth [8] claim that this is more common in patients in the fourth decade of life. We cannot discount the fact that the patient was 73 years old, and so the patient presents risk factors noted by Puvanendran et al. [2].

The diagnosis and treatment of NF should be established with the support of a multidisciplinary team before identifying the causative agent or the microbiota involved. Additionally, there is consensus that the elimination of the causative as well as the focus of the infection should be soon obtained to prevent further damage agent, since patients with NF are susceptible to rapid liquefaction in the progressive subcutaneous fat and connective tissue while the overlying skin is saved. Once the patient is not treated on time, the disease may progress rapidly to necrotizing mediastinitis and even death, which can occur in 10% to 40% of cases [9]. In the case reported the speed in establishing the diagnosis and initiating treatment resulted in the repair area having a decreased risk of sepsis and death.

Clinical and imaging findings can guide the dentist to the correct diagnosis of NF. The definitive diagnosis is made by surgical exploration, by the presence of necrosis of the fascia. However, the occurrence of local cyanosis and blistering yellowish or reddish dark content become present. Commonly, edema can be observed before other cutaneous signs appear. Other important aspects are palpable crepitation subcutaneous gas, as well as intense pain. The necrosis of the fascia is typically more extensive than suggested by the clinical aspect [10].

In the early stages, NF and cellulite are hardly distinguishable. In addition, NF and hemorrhaging blisters cellulite feature many other factors in common. Both are painful conditions, with potential to evolve quickly to necrosis and gangrene, which have the same predisposing factors and can have the same etiologic agents [10]. Clues to the diagnosis of NF include inelastic edema, cyanosis, pallor and hypoesthesia, crackling skin, muscle weakness, foul-smelling “dishwater” exudate, absence of lymphangitis, and rapid progression of the infection. Systemic manifestations of sepsis are usually present, including altered mental status, tachycardia, tachypnea, leukocytosis (>12000 mm^3^), fever, hypocalcemia, and metabolic acidosis. Bacteriological tests (direct and cultures) from the wound exudate, blister fluid, excised tissue, material aspirated tissue, and blood are essential for a proper diagnosis [11].

Disseminated intravascular coagulation and thrombocytopenia are common in any severe sepsis, and other hematological parameters should be interpreted with caution [12]. With a wide range of values reported in NF, the leucocyte count is less helpful for diagnosis. Acute renal failure is the norm in severe sepsis, and dosing of renal excreted antimicrobials should be adjusted accordingly. Bacterial infection, inflammation, thrombosis, and necrosis all increase serum C-reactive protein (CRP). Raised serum creatinine kinase (CK) indicates myositis or myonecrosis, as well as the effects of circulating toxins or ischemia [13, 14]. Involvement of adjacent muscle raises CK and is not present in all cases of NF, but CK levels of 600 U/L gave a sensitivity of 58% and a specificity of 95% for cases of NF. The most reliable indicators of underlying NF were found to be CRP, creatinine, hemoglobin, leucocyte count, sodium, and serum glucose [15].

X-ray is used to confirm the presence of gas in subcutaneous region as evidence of NF. However, unless the gas was confined more superficially, rarely could it be demonstrated for this examination. In contrast, CT provides the presence and extent of abnormal gas, in addition, to show necrosis with asymmetric thickening of the fascia. Walshaw and Deans reviewed the scans of 20 patients with NF, and 11 (55%) of them had gas in subcutaneous region [16]. CT also helps in differential diagnosis for evidence of myonecrosis, which suggests muscle impairment and conditions other than NF, in that such involvement is late and secondary [17]. MRI can also provide early diagnosis of NF and demonstrate the need for surgery and also determine the extent of involvement, thus facilitating presurgery [18].

Surgical treatment is fundamental to increasing the chance of patient survival. Combining surgery with multiple broad-spectrum antibiotics may be performed to combat NF. In this case, the antibiotic, surgical debridement, electrolyte replacement, and the use of drains were effective in fighting this infection. Treatment also involved a surgical Pen Rose drain installation for removal of necrotic tissue, tissue debridement, and irrigation. An antibiogram culture was made, as recommended [8]. Hyperbaric oxygen (HBO) therapy was another studied approach to treatment in the late 20th century. This adjunctive therapy is thought to increase tissue partial pressure of oxygen up to four times the normal, thus increasing bacterial killing and facilitating wound healing [19]. However, the few studies that have investigated HBO therapy in NF show little outcome benefit [20–22]. HBO is also believed to increase the bactericidal action of neutrophils since at low oxygen tensions peroxide-dependent killing mechanisms are less efficient [23]. Nonetheless, the overall evidence of benefit in nonclostridial NF is weak. Despite reports of rapid amelioration of clinical and mental status after only one HBO session, there are few published data to support the use of HBO in NF [7, 9, 10, 24]. In this case report such therapy was not applied.

Early thorough debridement is essential and produces large areas that need covering [25, 26]. Negative pressure therapy, vacuum-assisted closure dressing, with a continuous pressure of 40–100 mmHg is useful for wound coverage and encourages granulation before and after skin grafting [27, 28]. Although debridement was performed by the conventional technique, modern techniques such as “bear claw” are used for this purpose.

Yadav et al. [29] state that, generally, recovery of the surgical wound may take up to 150 days; in the case described, the wound was completely repaired in 50 days, indicating that the therapy came to a proper resolution. In this case report, after cicatrization, the patient presents substance loss due to repair by second intention requiring plastic surgical correction, which was refused by the patient.

Cervical NF in geriatric patient is rare, and even when establishing the diagnosis and having it timely treated, patient can suffer irreversible damage or even death. Therefore, the presentation of this clinical case can serve as guidance to dentists in order to maintain an aseptic chain and be aware of the clinical condition of older patients and the systemic conditions that may increase the risk of infections.
